# Mac-1 Directly Binds to the Endothelial Protein C-Receptor: A Link between the Protein C Anticoagulant Pathway and Inflammation?

**DOI:** 10.1371/journal.pone.0053103

**Published:** 2013-02-07

**Authors:** Katrin Fink, Hans-Jörg Busch, Natascha Bourgeois, Meike Schwarz, Dennis Wolf, Andreas Zirlik, Karlheinz Peter, Christoph Bode, Constantin von zur Muhlen

**Affiliations:** 1 Department of Cardiology and Angiology I, University Heart Center Freiburg, Freiburg, Germany; 2 Department of Emergency Medicine, University Hospital of Freiburg, Freiburg, Germany; 3 Baker IDI Heart and Diabetes Institute, Melbourne, Australia; UAE University, United Arab Emirates

## Abstract

**Objective:**

The endothelial protein C-receptor (EPCR) is an endothelial transmembrane protein that binds protein C and activated protein C (APC) with equal affinity, thereby facilitating APC formation. APC has anticoagulant, antiapoptotic and antiinflammatory properties. Soluble EPCR, released by the endothelium, may bind activated neutrophils, thereby modulating cell adhesion. EPCR is therefore considered as a possible link between the anticoagulant properties of protein C and the inflammatory response of neutrophils. In the present study, we aimed to provide proof of concept for a direct binding of EPCR to the β_2_ –integrin Mac-1 on monocytic cells under static and physiological flow conditions.

**Measurements and Main Results:**

Under static conditions, human monocytes bind soluble EPCR in a concentration dependent manner, as demonstrated by flow cytometry. Binding can be inhibited by specific antibodies (anti-EPCR and anti-Mac-1). Specific binding was confirmed by a static adhesion assay, where a transfected Mac-1 expressing CHO cell line (Mac-1+ cells) bound significantly more recombinant EPCR compared to Mac-1+ cells blocked by anti-Mac-1-antibody and native CHO cells. Under physiological flow conditions, monocyte binding to the endothelium could be significantly blocked by both, anti-EPCR and anti-Mac-1 antibodies in a dynamic adhesion assay at physiological flow conditions. Pre-treatment of endothelial cells with APC (drotrecogin alfa) diminished monocyte adhesion significantly in a comparable extent to anti-EPCR.

**Conclusions:**

In the present study, we demonstrate a direct binding of Mac-1 on monocytes to the endothelial protein C receptor under static and flow conditions. This binding suggests a link between the protein C anticoagulant pathway and inflammation at the endothelium side, such as in acute vascular inflammation or septicaemia.

## Introduction

The endothelial protein C-receptor (EPCR) is an endothelial transmembrane type 1 molecule [1] that is expressed primarily on large blood vessels [2]. Protein C (PC) binding to EPCR facilitates formation of activated protein C (APC), but EPCR binds PC and APC with equal affinity [3]. The PC pathway plays a key role in the regulation of blood coagulation by inhibiting thrombin generation [4], but also in limiting inflammatory response [3]. It is thought to decrease endothelial cell apoptosis in response to inflammatory cytokines and ischemia, thereby linking inflammation and endothelium [3], [5].

A soluble form of EPCR that can be released by the endothelium into circulation retains full ligand-binding ability [6]. Soluble EPCR (sEPCR) binds to activated neutrophils [7], and increased levels of sEPCR were found in patients with sepsis or systemic lupus erythematosus [8].

The slow inactivation of APC bound to EPCR by plasma protease inhibitors allows APC to signal cells. APC has been shown to have anticoagulant, anti-inflammatory and antiapoptotic activity at the cellular level [9], [10], [11]. In detail the APC-EPCR complex appears to be involved in cellular signalling mechanisms that down-regulate inflammatory cytokine formation [3], and APC has been demonstrated to block leukocyte adhesion in vivo, thereby reducing ischemia-reperfusion–induced injury [12]. Previously, recombinant human APC (drotrecogin alfa) has been shown to reduce the risk of death in patients with severe sepsis [13].

Adhesion molecules play a crucial role in vascular biology by mediating cell–cell and cell–matrix adhesion as well as by binding soluble ligands. The β_2_-integrin Mac-1 (CD11b/CD18) is expressed predominantly on monocytes, granulocytes and macrophages [14], and is known to interact with various ligands to serve different biological functions [15], [16], [17], [18], [19]. Mac-1 is known to mediate leukocyte adhesion to the vascular wall by binding to intercellular adhesion molecule-1 (ICAM-1) on endothelial cells, which, for example, is a precondition for chemotaxis-induced leukocyte extravasation [14], [20].

It was previously found that sEPCR binds to activated neutrophils via proteinase-3 and that this binding is partially dependent on Mac-1, suggesting a link between the protein C anticoagulant pathway and neutrophil functions [7]. Therefore, in the present study, we aimed to show a direct binding of EPCR to monocyte Mac-1 under static and physiological flow conditions, in order to identify another, so far unknown, binding partner of Mac-1. This interaction could be another link between vascular inflammation and coagulation in vascular inflammatory diseases, or in acute systemic inflammatory conditions such as septicaemia.

## Materials and Methods

### Cell culture of HUVECs

Human umbilical vein endothelial cells (HUVEC) were obtained from Promocell^TM^ (Heidelberg, Germany). The cells were cultured in endothelial cell growth medium advanced (Provitro, Berlin, Germany), containing 10% fetal calf serum (FCS), Heparin (22,50 µg), human recombinant epidermal growth factor (5 ng), human recombinant fibroblast growth factor (10 ng), human recombinant vascular endothelial growth factor (0,5 ng), human recombinant insulin-like growth factor-1 (20 ng), ascorbic acid (1 µg), hydrocortisone (0,20 µg), gentamicin (50 µg), L-glutamine (2 mmol) and cell culture plastic was from Nunc (Rolkilde, Denmark). Cultures were kept at 37°C in a 5% CO_2_ humidified atmosphere.

#### Mac-1 transfected CHO cells

Chinese hamster ovary (CHO) cell lines were generated expressing recombinant Mac-1 either in a native or a mutant form with a GFFKR deletion of the α-subunit (CD11b), which leads to the constitutive activation of Mac-1 and promotes ligand binding [21], [22]. CHO cells were maintained in modified Eagle medium (DMEM; Lonza, Verviers, Belgium) with 10% FCS, 100 U/mL penicillin, 100 μg/mL streptomycin, 1% L-glutamine, 1% non-essential amino acids, 700 μg/mL geneticin and 250 μg/mL zeocin [23].

#### Monocytes

After ethical approval from the ethics committee of our institution (10015/12), monocytes were isolated from buffy coat leukocytes or citrated human blood from healthy volunteers. Participants provided verbal informed consent to participate in this study. Monocytes were isolated by Ficoll (Biocoll Separating Solution, Biochrom, Berlin, Germany) gradient centrifugation and plastic adhesion.

Cells were mixed 1∶1 (in case of blood separation) or 1∶5 (in case of buffy coat separation) with phosphate buffered saline (PBS; Lonza, Veriers, Belgium). After centrifugation at 800× g for 20 min at room temperature the intermediate layer of cells was removed and washed with PBS.

Cells were maintained in RPMI medium (Invitrogen, Paisley, UK) with 10% FCS, 1% non-essential aminoacids, 2 mmol L-glutamine, 100 U/mL penicillin and 100 μg/mL streptomycin.

### Antibodies and reagents

The following fluorescence-labelled antibodies were used: anti-human CD11b-FITC from Becton Dickinson (San Jose, CA, USA), anti-human CD201 (EPCR)-PE from BD Biosciences, Pharmingen (Heidelberg, Germany) and anti-GST (26H1)-Alexa Fluor 488 from Cell Signaling Technology (Frankfurt, Germany). Mouse IgG_1_–FITC/PE (Beckman Coulter, Marseille, France) served as isotypic control.

The unlabeled antibody anti-Mac-1 (LEAF TM purified anti-Human CD11b, Clone ICRF44) was purchased from Biozol (Eching, Germany) and unlabeled anti-human CD201 (EPCR) from Becton Dickinson GmbH (Heidelberg, Germany). Vybrant^TM^ carboxyfluorescein-diacetate (CFDA; Invitrogen, Darmstadt, Germany) was used for staining of monocytes in flow chamber experiments. Recombinant soluble EPCR with N-terminal GST-tag was obtained from Abnova (Biozol, Eching, Germany), Phorbol 12-myristate 13-acetate (PMA) from Sigma (Taufkirchen, Germany). Activated protein C (drotrecogin alfa – Xigris^TM^) was a gift from Eli Lilly (RA Houten, Holland).

### Flow cytometry

Flow cytometric analysis was performed on a three-color flow cytometer (FACSCalibur^TM^, BD Biosciences) with individual settings for each antibody utilizing Cell Quest Pro^TM^ software (BD Biosciences). The mean fluorescence indices were analyzed employing the BD software.

#### Binding of sEPCR to monocytes and specific blockade

Isolated monocytes obtained from healthy volunteers were isolated as described above and, if necessary, stimulated with PMA (200 ng/mL) for 10 min. After a washing step, the cells were incubated with increasing concentrations of soluble recombinant, GST-tagged EPCR (6 µg/mL, 12 µg/mL, 24 µg/mL) for 30 min in the presence or absence of anti-EPCR (20 µL  = 0.5 mg/mL), or anti-Mac-1 (20 µL  = 1 mg/mL) antibody was added and incubated for 30 min. Bound sEPCR was detected by an Alexa Fluor-labeled antibody against the GST-tag. After washing with PBS and centrifugation with 500 rpm for 5 minutes, the pellet was resuspended in 300 µL Cellfix (Becton Dickinson) and analyzed on a FACScan (Becton Dickinson). Monocytes were identified on the forward/sideward scatter. Isotype IgGs served as controls.

### Static and Dynamic Adhesion Assays

#### Static adhesion assay

96-well plates (Nunc ImmunoPlate, MaxiSor^TM^) were coated with sEPCR (10 µg/ml) in PBS at 4°C overnight, blocked with 1% BSA (Serva Electrophoresis GmbH, Heidelberg, Germany) for 1 hour at room temperature, washed with PBS three times, and incubated with native (CHO) or transfected CHO cells with permanently activated Mac-1 (Mac-1+) at a density of 1×10^5^/mL cells per well. Cells were allowed to adhere for 45 min at 37°C, and adhesion was analyzed under static conditions in the presence or absence of anti-Mac-1 antibody (10 µg/mL). PBS was used to wash out unbound cells and washing steps were repeated until the negative control did not contain any more adhering cells. Analysis was performed by manually counting adhering cells. Plates coated with 1% BSA served as negative control, plates coated without blocking served as positive control.

#### Dynamic Adhesion Assay and endothelial blocking by activated protein C (APC)

HUVECs were grown in 35 mm dishes (Costar, Bethesda, MD), stimulated with TNFα (50 ng/mL) for 12–24 hours and were subjected to flow chamber. The Glycotech flow chamber (Gaithersburg, MD) was assembled with the dish as the bottom of the resulting parallel flow chamber. The chamber and tubes were filled with PBS prior to the experiment. Subsequently, isolated human monocytes, with or without stimulation with PMA (200 ng/mL) for 10 min, were applied with a syringe and shear stress was induced with a syringe pump (Harvard apparatus PHD2000, Holliston, MA) with a flow rate of 0.25 dyne/cm^2^ (venous flow) for a total of 10 min, and then with 15 dyne/cm^2^ (arterial flow) for 1 min. Monocytes were allowed to adhere to the endothelial cell layer after either pre-treatment of monocytes with anti-Mac-1 antibody (60 µg/1×10^6^ monocytes), incubation of HUVECs with anti-EPCR (10 µg/mL cell medium per dish), both antibodies, or without blocking antibody. In a second approach, pre-treatment of HUVECs with drotrecogin alfa in different concentrations (1 µg/mL, 5 µg/mL, 10 µg/mL) was compared to anti-EPCR-blocking. Adherent cells were quantified under the microscope and monocytes were visualized by CFDA staining (500 µL CFDA per 70*10?6 monocytes, incubated for 15 min at 37°C in a waterbath). Data from at least four different experiments were analyzed.

## Results

### Binding of sEPCR to monocytes

**Figure 1:**
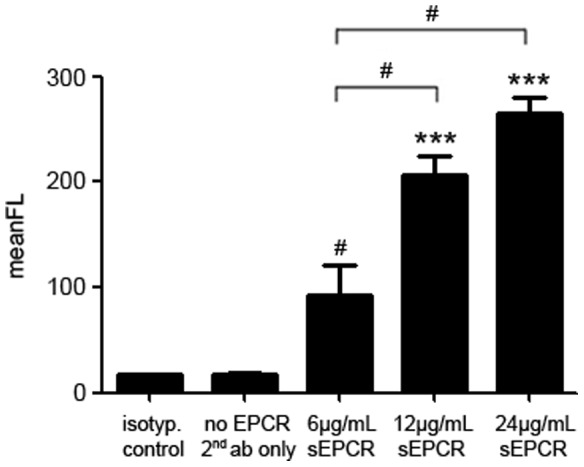
Monocytes bind sEPCR in a concentration dependent manner. Monocytes bind soluble, recombinant EPCR in a concentration dependent manner in flow cytometry analysis. GST tagged-EPCR was detected with an Alexa Flour-labeled anti-GST antibody. Secondary antibody and isotype IgG served as controls (black bars on the left and second from left). (*** *p*<0.0001; # *p*<0.05 vs. controls).

First we addressed the direct binding of soluble, recombinant sEPCR to freshly isolated human monocytes. In flow cytometric analysis, monocytes are able to bind sEPCR in a concentration dependent manner (Fig. 1).

**Figure 2:**
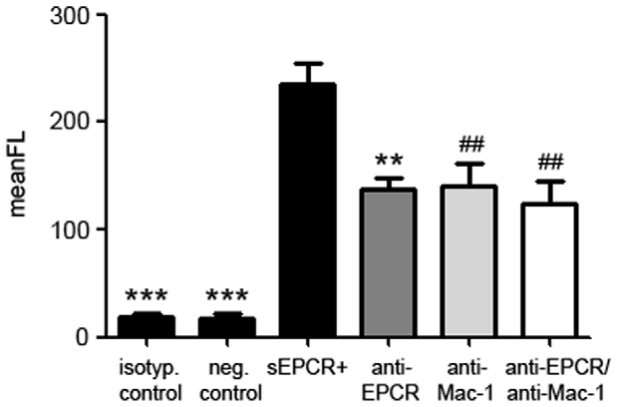
Blockade of sEPCR binding to monocytes by anti-EPCR and anti-Mac-1 in flow cytometry. Binding of soluble, recombinant EPCR to monocytes (black bar second from left) can be blocked by anti-EPCR (dark grey bar), by anti-Mac-1 (light grey bar) and by both antibodies (white bar) to an equal extend. Monocytes without addition of sEPCR and isotype IgG served as negative controls (black bars on the left and second from left). (*** *p*<0.0001; ** *p*<0.001; ^##^ *p*<0.005 vs. sEPCR+).

Specific binding of a flag-tagged recombinant sEPCR to non-stimulated and PMA-stimulated human monocytes was evaluated by flow cytometry in the presence or absence of anti–Mac-1 and anti-EPCR antibody. Binding blockade by anti-Mac-1 and anti-EPCR antibodies inhibited this binding to an equal extend (Fig. 2).

### Specific binding of Mac-1 to EPCR in static adhesion assay

**Figure 3:**
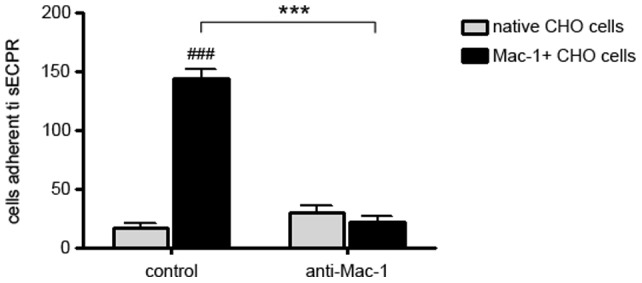
Specific binding of CHO cells expressing activated Mac-1 to recombinant EPCR in static adhesion assay. Specific binding of CHO cells transfected with permanently activated Mac-1 (Mac-1+ CHO cells; black bars) to soluble, recombinant EPCR in a static adhesion assay (left). Blocking with an anti-Mac-1 antibody resulted in loss of EPCR binding capacity of Mac-1+ CHO cells (right). Native CHO cells without transfection of Mac-1 served as a negative control (light grey bars). (^###^ *p*<0.0005; ns  =  statistically not significant vs.native CHO cells; *** *p*<0.0001).

Specific binding and the functional relevance of the interaction between EPCR and Mac-1 for monocyte adhesion was tested by a static adhesion assay. To verify that Mac-1 can bind to sEPCR, we used a transfected CHO cell line that provided clearly defined states of Mac-1 affinity as well as control cells without any Mac-1 expression. Incubation of sEPCR-coated plates with native CHO cells, or CHO cells transfected with permanently activated Mac-1 (Mac-1+) confirmed the findings obtained with monocytes. CHO cells expressing the activated Mac-1 (Mac-1+) displayed significantly enhanced adhesion to immobilized EPCR compared to native CHO cells and this effect could be reversed by an anti–Mac-1 antibody (Fig. 3).

### Specific binding of Mac-1 to EPCR under flow conditions

In a second approach, we tested whether EPCR–Mac-1 interaction persists under flow conditions, similar to those in human vessels. Under venous flow conditions, monocytes adhered on HUVECs with a maximum after 10 min. Monocyte adhesion was slightly attenuated but persisted under simulated arterial flow conditions, suggesting tight binding of most of the monocytes.

**Figure 4:**
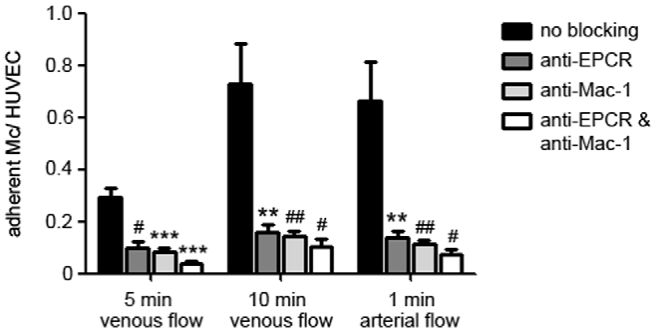
Blockade of monocyte binding to endothelial cells by anti-EPCR and anti-Mac-1 under flow conditions. Monocyte binding to HUVECs in a dynamic adhesion assay after 5 min (left) and 10 min venous flow (middle), and after 1 min arterial flow (right). When EPCR was blocked on HUVECs (dark grey bars), Mac-1 on monocytes (light grey bars), or both (white bars) monocyte binding could be significantly diminished compared to native cells without antibody treatment (black bars). (*** *p*<0.0001; ** *p*<0.001; ^##^ *p*<0.005; ^#^ *p*<0.05 vs. no blocking).

Pre-treatment of monocytes with anti-Mac-1 antibody and incubation of HUVECs with anti-EPCR antibody, both and each antibody alone significantly attenuated monocyte adhesion at all time points and even under arterial flow conditions (Fig. 4). These data support the concept of direct EPCR–Mac-1 interaction that mediates leukocyte adhesion (Fig. 4).

### Blockade of monocyte-endothelial interaction by activated protein C

**Figure 5:**
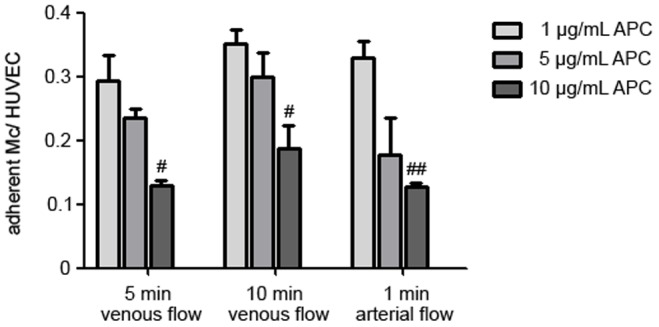
Blockade of monocyte binding to endothelial cells by APC in different concentrations under flow conditions. Monocyte binding to HUVECs in dynamic adhesion assays after 5 min (left) and 10 min venous flow (middle), and after 1 min arterial flow (right). Pre-treatment of HUVECs with 10 µg/mL activated protein C (drotrecogin alfa) (dark grey bars) diminished monocyte adhesion compared to 1 µg/mL (light grey bars) and 5 µg/mL (grey bars) drotrecogin alfa. (^##^ *p*<0.005; ^#^ *p*<0.05 vs. 1 µg/mL APC).

**Figure 6:**
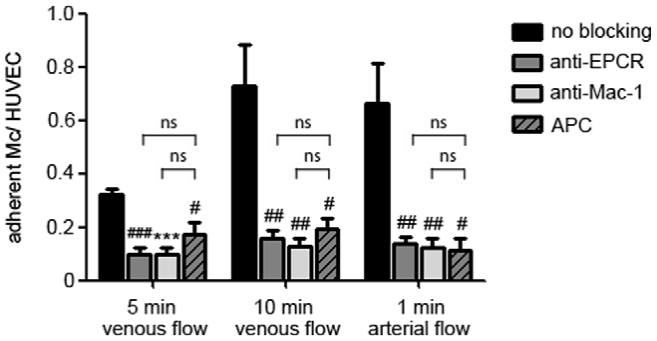
Equal blocking of monocyte binding to endothelial cells by anti-EPCR and APC under flow conditions. Monocyte binding to HUVECs in dynamic adhesion assay after 5 min (left) and 10 min venous flow (middle), and after 1 min arterial flow (right). Pre-treatment of HUVECs with anti-EPCR (dark grey bars), anti-Mac-1 (light grey bars), or with activated protein C (drotrecogin alfa; crosshatched bars) diminished monocyte adhesion to an equal extent compared to control. (*** *p*<0.0001; ^###^ *p*<0.0005; ^##^ *p*<0.005; ^#^ *p*<0.05 vs. no blocking; ns  =  statistically not signifiant).

Pre-treatment of HUVECs with increasing concentrations of drotrecogin alfa diminished monocyte adhesion in a concentration dependent manner with a maximum at 10 µg/mL (Fig. 5). APC inhibits monocyte adhesion on endothelial cells to a similar extent as anti-EPCR, or anti-Mac-1 antibodies (Fig. 6).

## Discussion

The current data demonstrate that Mac-1 on monocytes binds directly to the endothelial protein C receptor. Direct binding was identified by static adhesion assay and indirectly by blockade of the binding partners by specific antibodies in flow cytometry, as well as in a dynamic adhesion assay. Furthermore, activated protein C diminished monocyte adhesion under flow conditions comparable to the specific anti-EPCR antibody, suggesting a competitive receptor blockade in this setting.

The protein C pathway serves as a major system for controlling thrombosis, limiting inflammatory responses and potentially decreasing endothelial cell apoptosis in response to inflammatory cytokines and ischemia [3]. The essential components of the pathway involve the EPCR, protein C, protein S, thrombin and thrombomodulin [3]. EPCR is expressed constitutively on endothelial cells and its soluble form has been found in medium of cultured human endothelial cells [24]. Soluble EPCR is known to bind to activated neutrophils and elevated levels of sEPCR were also found in patients suffering from septicemia [8].

In our study, we could demonstrate that sEPCR binds to human monocytes in a concentration-dependent manner. Since PMA-stimulated monocytes express more Mac-1 (CD11b/CD18) on their surface and are able to bind more sEPCR [8], one might assume a direct interaction of these receptors.

Kurosawa et al. hypothesized that Mac-1 contributes to sEPCR-binding towards activated neutrophils in a process that involves binding directly to leukocyte-derived proteinase 3 [7]. Interaction of EPCR with leukocytes is in this context another observation, linking the receptor to the regulation of the inflammatory response [3]. Therefore, we aimed to proof this direct binding in the present study. Under static conditions, binding could already be assumed, since sEPCR-binding to monocytes could be blocked by both, anti-EPCR- and anti-Mac-1-antibodies. Proof of direct binding was performed by applying a static adhesion assay. To evaluate the importance of this interaction, we tested physiological flow conditions using a dynamic adhesion assay. Accordingly, binding of monocytes to the endothelium could be blocked by both, anti-EPCR- and anti-Mac-1-antibodies, pointing out the relevance of the binding characteristics in vivo. In contrast, Esmon et al. postulated, that soluble EPCR may block tight attachment of leukocytes by binding to Proteinase 3 and its complexes with Mac-1 on activated neutrophils [3], [7], but in our model we can only make a statement of EPCR constitutively expressed on the endothelium.

APC limits leukocyte adhesion to the endothelium and extravasation into tissues [12], [25], inhibits the release of inflammatory cytokines [25], [26] and other inflammatory events, such as NF-κB nuclear translocation, or expression of adhesion molecules in endothelial cells [10], [27]. It is suggested to play a role as an endothelial cell or microvascular modulator with properties in opposition to proinflammatory cytokines [28]. Recombinant human APC (drotrecogin alfa) has been shown to protect patients with severe sepsis and was therapeutically effective in ameliorating experimental colitis [29]. Accordingly, in the present study, we could demonstrate blocking of monocyte adhesion to the endothelium by drotrecogin alfa under physiological flow conditions in a concentration dependent manner. Since our data demonstrate an equal blocking of monocyte adhesion to the endothelium by APC and by anti-EPCR, we postulate that EPCR itself plays a role in leukocyte adhesion to the endothelium, probably in part via binding to Mac-1.

A limitation of the present study is the lack of in vivo data to support the hypothesis that the interaction is indeed relevant. Further studies are needed to test the effect of EPCR blockade, or EPCR-deficiency with respect to endothelial-leukocyte interaction.

## Conclusions

In this study we demonstrate a direct binding of monocyte Mac-1 to the endothelial protein C receptor in human cells under static and flow conditions. This interaction may play a role in linking vascular inflammation and coagulation in acute vascular inflammatory diseases or septicaemia. Therefore, inhibition of this interaction (e.g. by administration of APC) may have therapeutic implications that need to be addressed in further studies.
